# BARRIERS AND BENEFITS TO TECHNOLOGY ADOPTION BY DEMENTIA CAREGIVERS

**DOI:** 10.1093/geroni/igae098.2579

**Published:** 2024-12-31

**Authors:** Patti Simone, Julia Scott, Sheila Yueter, Emma Cepukenas, McKenzie Himes, Kennedy Anderson, An Mai, Kiren Grewal

**Affiliations:** Santa Clara University, Santa Clara, California, United States; Santa Clara University, Santa Clara, California, United States; Santa Clara University, Santa Clara, California, United States; Santa Clara University, Santa Clara, California, United States; Santa Clara University, Santa Clara, California, United States; Santa Clara University, Santa Clara, California, United States; Santa Clara University, Santa Clara, California, United States; Santa Clara University, Santa Clara, California, United States

## Abstract

Technology can support caregivers of people with dementia but to date, they are not adopted at scale. We surveyed a range of caregivers regarding their use of technology they use to assist in caregiving tasks to better understand the relationship between technological tool use and satisfaction with caregiving. An anonymous online survey was distributed to the local community and dementia care networks in California and Washington (N = 69) and a subset of the respondents were invited to engage in a didactic interview (N = 19). Respondents identified the most important unmet need for caregivers was access to resources (53%), such as healthcare navigation, mental health support, and financial planning, all of which could be improved with technological tools. Furthermore, the majority of caregivers (64%) reported significant emotional struggle and conflict associated with their role. Similarly, interviews with professionals and caregivers illustrated concerns about caregiver well-being. Even though three-quarters of survey respondents reported using some technological tools to assist caregiving (e.g., medication and routine tracking applications, mobility assistive devices, sensors and monitors), the study showed that caregivers felt inadequately supported and they relied minimally on technology to address their pain points (e.g., mental health). Instead they noted barriers of access and usability of products as limiting features of adopting additional technology. Effective use of digital technologies can reduce caregiver stress by simplifying care tasks and delivering more convenient access to support. Strategies in product design and user communication that engage dementia care partners would likely accelerate adoption of technologies.

